# Application of dandelion-like Sm_2_O_3_/Co_3_O_4_/rGO in high performance supercapacitors†

**DOI:** 10.1039/d3ra06352f

**Published:** 2024-01-09

**Authors:** Yanling Lv, Shixiang Lu, Wenguo Xu, Yulin Xin, Xiaoyan Wang, Shasha Wang, Jiaan Yu

**Affiliations:** a School of Chemistry and Chemical Engineering, Beijing Institute of Technology Beijing 100081 China shixianglu@bit.edu.cn +86 10 68912631 +86 10 68912667

## Abstract

Novel 2D material-based supercapacitors are promising candidates for energy applications due to their distinctive physical, chemical, and electrochemical properties. In this study, a dandelion-like structure material comprised of Sm_2_O_3_, Co_3_O_4_, and 2D reduced graphene oxide (rGO) on nickel foam (NF) was synthesised using a hydrothermal method followed by subsequent annealing treatment. This dandelion composite grows further through the tremella-like structure of Sm_2_O_3_ and Co_3_O_4_, which facilitates the diffusion of ions and prevents structural collapse during charging and discharging. A substantial number of active sites are generated during redox reactions by the unique surface morphology of the Sm_2_O_3_/Co_3_O_4_/rGO/NF composite (SCGN). The maximum specific capacity the SCGN material achieves is 3448 F g^−1^ for 1 A g^−1^ in a 6 mol L^−1^ KOH solution. Benefiting from its morphological structure, the prepared composite (SCGN) exhibits a high cyclability of 93.2% over 3000 charge–discharge cycles at 10 A g^−1^ and a coulombic efficiency of 97.4%. Additionally, the assembled SCGN//SCGN symmetric supercapacitors deliver a high energy density of 64 W h kg^−1^ with a power density of 300 W kg^−1^, which increases to an outstanding power density of 12 000 W kg^−1^ at 28.7 W h kg^−1^ and long cycle stability (80.9% capacitance retention after 30 000 cycles). These results suggest that the manufactured SCGN electrodes could be viable active electrode materials for electrochemical supercapacitors.

## Abstract 2

A dandelion-like structure composite was synthesised through a facile hydrothermal method and annealing treatment, exhibiting excellent electrode performance for supercapacitors.

## Graphical abstract

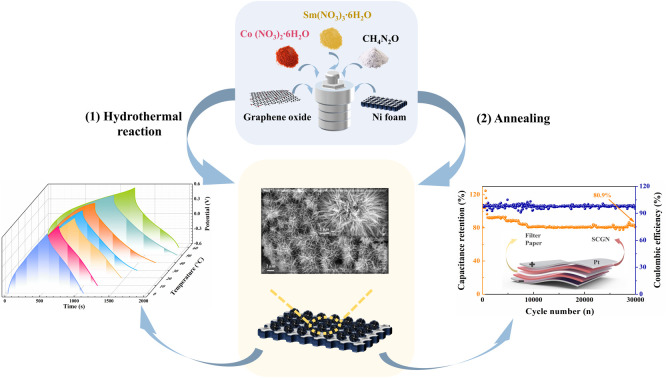

## 1. Introduction

Growing concerns about fossil fuel depletion and environmental pollution have spurred the study of efficient, clean, and sustainable energy sources, along with the development of new energy conversion and storage technologies.^1–3^ Recently, high-tech energy storage devices like supercapacitors have gained prominence as effective strategies to transition from a carbon-based energy economy to one predominantly fueled by renewables.^4–6^ Supercapacitors are attracting significant interest due to their high-power density, rapid charging, lengthy cycle life, and remarkable reversibility.^7–9^ Applications in portable electronics, hybrid electric vehicles, and astrovehicles are made possible by the appealing properties of supercapacitors.^10^ Nevertheless, the energy density of supercapacitors often falls short compared to lithium-ion batteries, restricting their commercial application.^11^

As we know, enhancing the capacitance of electrode material is a pivotal approach to increasing the energy density of supercapacitors. Hence, developing electrode material with high capacitance is one of the crucial challenges for supercapacitors, and Co_3_O_4_ and other pseudocapacitors based on transition metal oxides (TMOs) are anticipated to become primary electrochemically active materials.^12–14^ In the meantime, TMOs, combining the benefits of low cost, environmental friendliness, acceptable stability, high intrinsic activity, and theoretical specific capacitances, are anticipated to become a promising alternative to ones used in commerce.^15,16^ Although TMOs have been the subject of much investigation, the promise of creating high-performance supercapacitors is sadly hampered by the constraints of their oxidation active sites and the accompanying valence changes during redox processes.^17^ In this case, it is imperative to introduce another metal element to prepare multi-component metal oxides for optimising the overall performance of the system.

Rare earth (RE), often referred to as “modern industrial vitamins”, is a precious strategic resource.^18^ Different from the transition metal elements, rare earth (RE) elements have unique ground-state electronic configurations, and the unpaired 4f orbital electrons endow RE elements with many exceptional properties, such as optical, magnetic, and electrical properties.^19,20^ The distinctive ground-state electronic structure of RE elements facilitates the generation of stable RE ions with varying valence states, enabling charge storage through rapid transitions between these states. Recent years have witnessed major progress in multi-component metal oxide research, especially involving the utilisation of RE metals, as evinced by numerous research articles and technical reports. Xu *et al.*^21^ prepared Yb-doped α-Ni(OH)_2_ by co-precipitation method, achieving a discharge specific capacity of 295.1 mA h g^−1^ at 0.2 A g^−1^, surpassing that of the undoped material by 60 mA h g^−1^. Luo *et al.*^22^ prepared rare-earth CeO_2_ doped silver-ear Co_3_O_4_ through a hydrothermal process, and the specific capacitance at a current density of 1 A g^−1^ can be as high as 2260.8 F g^−1^.

Among rare earth oxides, samarium oxide (Sm_2_O_3_) is an essential c-structured rare earth oxide material extensively studied for its valence variability and high stability.^23,24^ In terms of energy band structure, Sm_2_O_3_ boasts a broad energy band gap of 5.1 eV and functions as a p-type metal oxide semiconductor, showing potential for applications in solar cells, nanoelectronics, semiconductor gases, and biochemical sensors.^25–28^ Despite these promising properties, Sm_2_O_3_ is insufficient for electrode material applications due to its inherently low electrical conductivity. To harness the advantages of each component fully, considering other high surface area carbon-based materials as electrode components becomes essential for achieving a larger potential window and improved conductivity.^29^ In the past decades, 2D layered materials have gradually become the focus of materials science by virtue of their unique layered structural properties and good optoelectronic properties.^30–35^ Reduced graphene oxide (rGO), as one of the 2D materials, stands out for supercapacitor applications due to its remarkable electrochemical properties, high surface area, and excellent electronic properties.^36–40^ Thus, carbon-based (such as rGO, porous carbon (PC), MXene, *etc.*) materials are used as pseudocapacitors in combination with metal oxides or conductive polymers.^41–45^ In the early literature, different capacitance values were obtained for graphene-based supercapacitors.^46^ In organic electrolytes, the specific capacitance value is 99 F g^−1^, and low-aggregated reduced graphene achieves an energy density of 28.5 W h kg^−1^ with a specific capacitance of 205 F g^−1^ in aqueous electrolytes.^47^

In this work, a facile one-step hydrothermal and annealing technique was used to create dandelion-like nanocomposites of Sm_2_O_3_/Co_3_O_4_/rGO on nickel foam (NF) (SCGN). It is expected that the introduction of Sm_2_O_3_ serves to compensate for the poor cycling stability of Co_3_O_4,_ while the addition of rGO addresses its poor electrical conductivity. This approach maximises the synergistic effects between various materials. The resulting composites underwent comprehensive analysis of their structure, morphology, and electrochemical properties. The SCGN electrodes exhibit excellent electrochemical properties, including high specific capacity (maximum specific capacitance of 3448 F g^−1^ at a current density of 1 A g^−1^) and robust cycling stability. High-performance symmetric supercapacitors with reliable energy storage and power output have been achieved using SCGN electrodes. The exceptional electrochemical performance of composite materials can be attributed to the synergistic effects between rGO and tightly anchored Sm_2_O_3_/Co_3_O_4_ nanoparticles on the nickel foam.

## 2. Experimental

### 2.1 Reagents and materials

Graphite powder was obtained from Shanghai Macklin Biochemical Technology Co., Ltd. Sulfuric acid (H_2_SO_4_, 98%), potassium permanganate (KMnO_4_), and hydrogen peroxide (H_2_O_2_, 30 wt%) were sourced from Xilong Scientific Co., Ltd. Hydrogen acid (HCl) and acetone (C_3_H_6_O) were provided by Tianjin Fuyu Fine Chemical Co., Ltd. Ethanol (C_2_H_5_OH, 99.5%) and potassium hydroxide (KOH) were purchased from Anhui Senrise Technology Co., Ltd. Potassium nitrate (KNO_3_) was purchased from Beijing Beihua Fine Chemical Co., Ltd. Urea (CH_4_N_2_O), cobalt nitrate hexahydrate (Co(NO_3_)_2_·6H_2_O) and samarium nitrate hexahydrate (Sm(NO_3_)_3_·6H_2_O) were obtained from Shanghai Aladdin Biochemical Technology Co., Ltd. Nickel foam (NF) was made available by Kunshan Tonghui Electronic Technology Co., Ltd. All chemicals used were of analytical purity, and deionised water was used in all experiments.

### 2.2 Preparation of Sm_2_O_3_/Co_3_O_4_/rGO/NF composite

#### 2.2.1 Pretreatment of conductive substrate NF

To eliminate any potential impurities, the nickel foams (NFs) (20 mm × 10 mm × 1 mm) were manually cut and subsequently subjected to successive washing with acetone, ethanol, and deionised water for 30 minutes in an ultrasonic water bath and then dried in an oven at 60 °C for 12 h for future use.

#### 2.2.2 Preparation of Sm_2_O_3_/Co_3_O_4_/rGO/NF

Initially, graphene oxide (GO) was prepared from graphite powder using the modified Hummers' method.^48^ In a typical procedure, cobalt nitrate hexahydrate, samarium nitrate hexahydrate, and urea were dissolved in deionised water and stirred for 1 h. The resulting suspension was then add-mixed with a GO suspension, which had been sonicated 2 h earlier. The above solutions were combined under vigorous stirring for 1 h. Afterwards, the obtained dark brown solution was transferred and sealed in a 25 mL stainless-steel Teflon autoclave placed with pretreated nickel foam. The mixture was heated to 180 °C for 9 h. After cooling to room temperature, the resulting product of Sm_2_O_3_/Co_3_O_4_/rGO/NF precursors underwent numerous washes with distilled water and ethanol. In this way, the collected dark blue samples were formed, which were subsequently dried in an oven at 60 °C for 8 h.

**Fig. 1:**
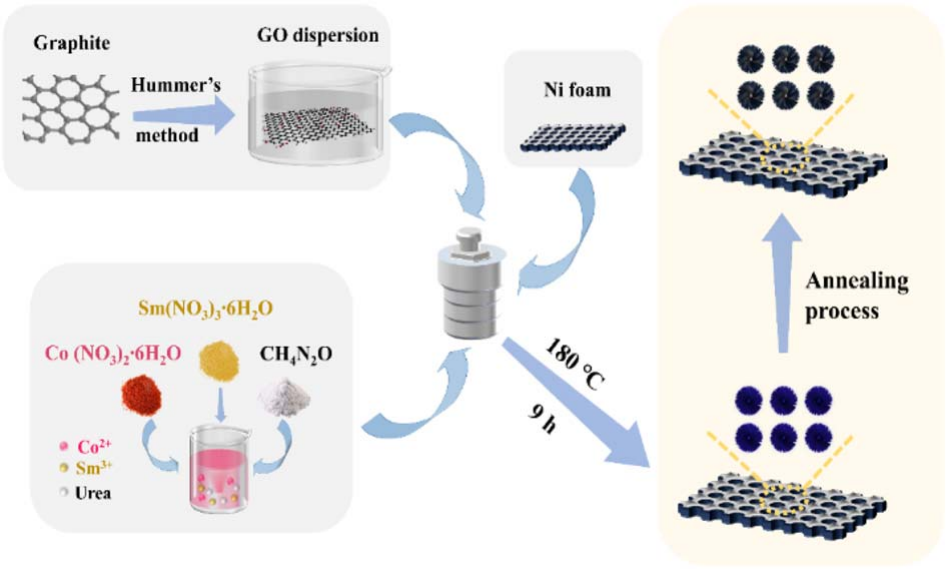
The preparation process of SCGN composite electrodes.

Finally, the dried precursors were calcined in a muffle furnace and annealed at 300 °C for 2 h to obtain Sm_2_O_3_/Co_3_O_4_/rGO/NF composite (SCGN). Fig. 1 illustrates the synthesis process of the SCGN. It is noteworthy that four more samples were prepared using the above similar synthesis route: Sm_2_O_3_/Co_3_O_4_/NF composite (SCN), Co_3_O_4_/rGO/NF composite (CGN), Sm_2_O_3_/rGO/NF composite (SGN) and rGO/NF composite, as shown in S1–S4 (ESI†).

### 2.3 Characterizations

The crystallinity of the materials was assessed using a Bruker D8 Advance X-ray powder diffractometer (XRD) equipped with Cu Kα (*λ* = 0.15418 nm) at 2*θ* angles ranging from 10° to 80°. The surface morphological structure of the samples was visualised using a scanning electron microscope (SEM, Quanta 600, FEI) with its energy spectrometer (EDS, Oxford, Gemini 300), as well as a high-resolution transmission electron microscope (HRTEM) and a transmission electron microscope (TEM, JEM-2100, JEOL). A Renishaw InVia confocal Raman spectrometer, a Leica DMLM microscope, and an argon ion laser (514.5 nm, model Stellar-REN, Modu-Laser) as the excitation source were used to capture the Raman spectra. Furthermore, X-ray photoelectron spectroscopy (XPS) measurements were performed using a PHI 5300 model instrument (Physical Electronics, USA). The surface area was determined *via* N_2_ adsorption and desorption tests using the Brunauer–Emmett–Teller (BET) apparatus, while the Barrett–Joyner–Halenda (BJH) method computed the pore size distribution.

### 2.4 Electrochemical characterisation

The surface electrochemical measurements, including cyclic voltammetry (CV), galvanostatic charge–discharge (GCD) performance, and electrochemical impedance spectroscopy (EIS), were carried out on CHI-760E electrochemical working station using a standard workstation cell in a 6 mol L^−1^ KOH electrolyte at ambient temperature. The prepared composite materials served as the working electrode, while a saturated calomel electrode (SCE) and a platinum electrode (20 mm × 10 mm) were used as the reference and counter electrodes, respectively. The CV curves were carried out at various scanning rates (5–100 mV s^−1^), and the GCD tests were performed within a potential window from −0.1 V to 0.4 V, encompassing different current densities ranging from 0.5 A g^−1^ to 10 A g^−1^. EIS was acquired with a perturbation amplitude of 5 mV at open circuit potential in a frequency range from 100 kHz to 0.01 kHz. The specific capacitance of the electrodes at different current densities can be calculated by using the corresponding equation:^49^1
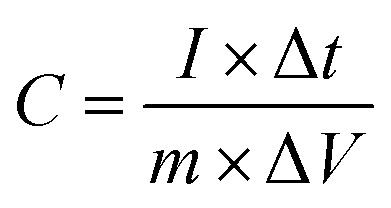
where *C* (F g^−1^) denotes the specific capacitance, *I* represents the discharge current (A), Δ*t* signifies the discharge time (s), Δ*V* stands for the voltage interval (V), and *m* is the mass of the active material (g).

To assess energy density and power density, the subsequent formulas were applied:2
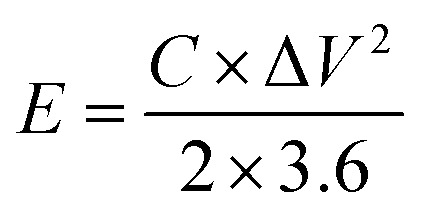
3
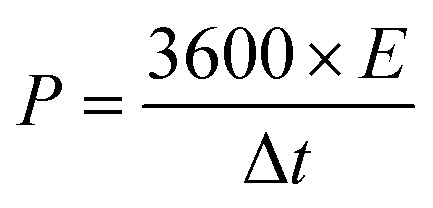
Here, *E* represents the specific energy (W h kg^−1^), *C* signifies the specific capacitance of SCGN (F g^−1^), and Δ*V* (V) is the potential window. *P* indicates the power density (W kg^−1^), and Δ*t* is the discharge time (s).

## 3. Result and discussion

### 3.1 Structural analysis

**Fig. 2:**
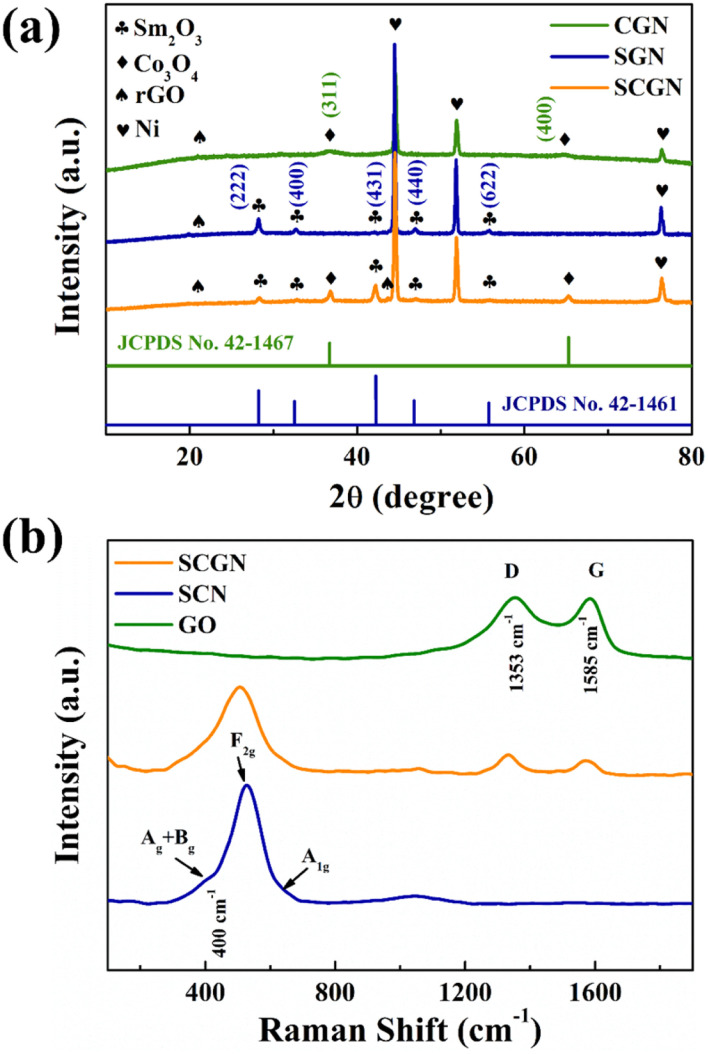
(a) XRD patterns of CGN, SGN, and SCGN; (b) Raman spectra of GO, SCN, and SCGN.

The phase structures of SCGN, SGN, and CGN were characterised through X-ray diffraction (XRD) analysis using Cu Kα radiation, as depicted in Fig. 2a. The confirmation of SCGN, SGN, and CGN formation relied on comparing diffraction peaks with the standard diffraction patterns of the respective material compound. The diffraction peaks of 2*θ* at 28.3°, 32.7°, 42.1°, 47.0° and 55.7° from the XRD pattern of SGN assign to (222), (400), (431), (440), and (622) crystal planes of cubic Sm_2_O_3_. This observation concurs with established literature findings.^50^ Moreover, the result is in good agreement with JCPDS card no. 42-1461. Notably, the prominent diffraction peak alignment between the SCGN and SGN patterns validates the successful anchoring of Sm_2_O_3_ particles onto the rGO sheets. All major diffraction peaks at 2*θ* values (36.8°, 65.2°) from the XRD pattern of CGN can be indexed to the (311) and (440) crystal planes of the cubic Co_3_O_4_ phase (JCPDS card no. 42-1467), respectively. In addition, the distinct and sharper diffraction peaks of Sm_2_O_3_ and Co_3_O_4_ underscore the composite's exceptional crystallinity. The XRD patterns of SCGN, SGN, and CGN further exhibit three robust intensity peaks at 44.5°, 51.8°, and 76.4°, corresponding to the (111), (200), and (220) crystal planes of metallic nickel. Notably, the XRD pattern of rGO exhibits two peaks at about 23.3° and 42.8° of 2*θ* values associated with (002) and (100) diffraction planes, respectively, albeit with faint intensity.

Raman of SCGN, SCN, and GO were subsequently performed to ascertain graphene's presence on SCGN. The comparative Raman spectra of SCGN and SCN were tested. Fig. 2b shows the comparative Raman spectra of GO, SCGN, and SCN in the 0–4000 cm^−1^ range. It can be seen in the obtained spectra that both GO and SCGN show two characteristic peaks at 1353 cm^−1^ and 1585 cm^−1^, corresponding to the D peak and G peak, respectively.^51^ Among them, the D peak is the characteristic peak of sp^3^ defects in carbon atoms, representing the defects and amorphous structures at the edges of graphene. The G peak pertains to the E_2g_ vibration of carbon atoms in graphene sp^2^ hybridisation, indicative of its structural stability.^52^ The ratio between the D-band and the G-band intensity (*I*_D_/*I*_G_) is a gauge of disorder or defect density, with higher ratios indicating more irregularity in rGO.^53^ Notably, the *I*_D_/*I*_G_ value of rGO within SCGN is 1.04, which is higher than the corresponding value of GO (0.99). It indicates that part of GO has been successfully converted to rGO during the reduction process. Electronic interactions between Sm_2_O_3_/Co_3_O_4_ and rGO contributed to heightened sp^2^ hybrid structures with increased defect density, fostering material disorder. The intensity peak at 400 cm^−1^ can be attributed to the combination of A_g_ and B_g_ vibrations of Sm_2_O_3_.^54^ The faint spectral band at 651 cm^−1^ is connected to the characteristic octahedral lattice position occupied by Co^3+^, which is linked to the A_1g_ symmetry, while the F_2g_ vibrational mode of Co_3_O_4_ is assigned to the 502 cm^−1^ band.^55,56^ These outcomes collectively confirm the successful preparation of the SCGN electrode material.

**Fig. 3:**
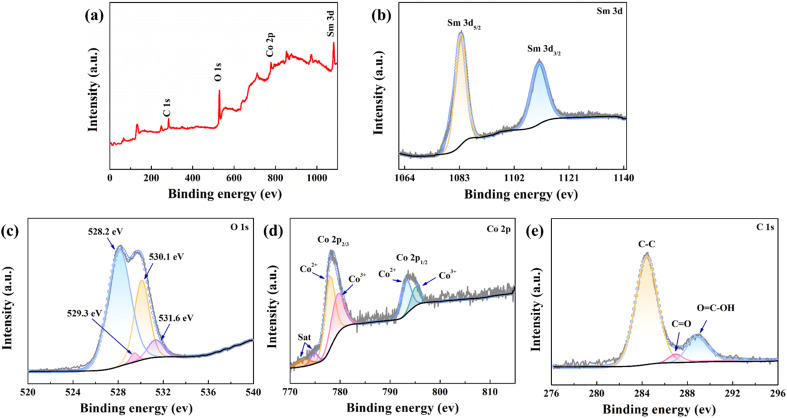
(a) XPS spectra of the SCGN composite; high-resolution XPS spectra of (b) Sm 3d, (c) O 1s, (d) Co 2p, (e) C 1s.

The valence state, oxidation state, and purity of the prepared SCGN electrode material were determined by the X-ray photoelectron (XPS) technique. The wide range XPS survey spectrum of the synthesised sample shown in Fig. 3a indicates no impurities in this nanocomposite. The deconvoluted Sm 3d spectrum (Fig. 3b) comprises two intense bands, 1083.3 eV and 1110.6 eV, corresponding to Sm 3d_5/2_ and Sm 3d_3/2_ valence states, respectively.^57^ At varied binding energies of O 1s, four peaks at 528.2 eV, 529.3 eV, 530.1 eV, and 531.6 eV correspond to the metal–oxygen bond in Fig. 3c. Specifically, the peak at 531.6 eV can be attributed to the oxygen of Sm^3+^–O groups, whereas another weaker peak with a binding energy of 529.3 eV is assigned to the oxygen of Sm^2+^–O groups, which is related to the oxygen in the crystal lattice of Sm_2_O_3_.^58^ The coexistence of these peaks reveals the presence of Sm in two different ionic states; thus, the oxygen vacancy in the crystal lattice of Sm_2_O_3_ aids in charge transfer. The Co 2p spectrum (Fig. 3d) is composed of two spin–orbit doublet characteristics (Co^2+^ and Co^3+^) and two shakeup satellites. The peak around 780.4 eV is indexed to Co^3+^, while the peaks positioned at 779.6 eV and 795.0 eV in the high-resolution XPS of Co 2p are ascribed to the Co 2p_3/2_ and Co 2p_1/2_ features of Co_3_O_4_.^59–61^ Additionally, Fig. 3e depicts the core level spectra of C 1s, revealing two extremely strong lines at 284.4 eV and 289 eV. These specific energy levels are ascribed to the C–C and O=C–OH groups.^62^ The weaker peak at 287 eV is designated for C=O (epoxy/ether).^63^ The results show the successful reduction of GO into rGO.

### 3.2 Morphology and microstructures

**Fig. 4:**
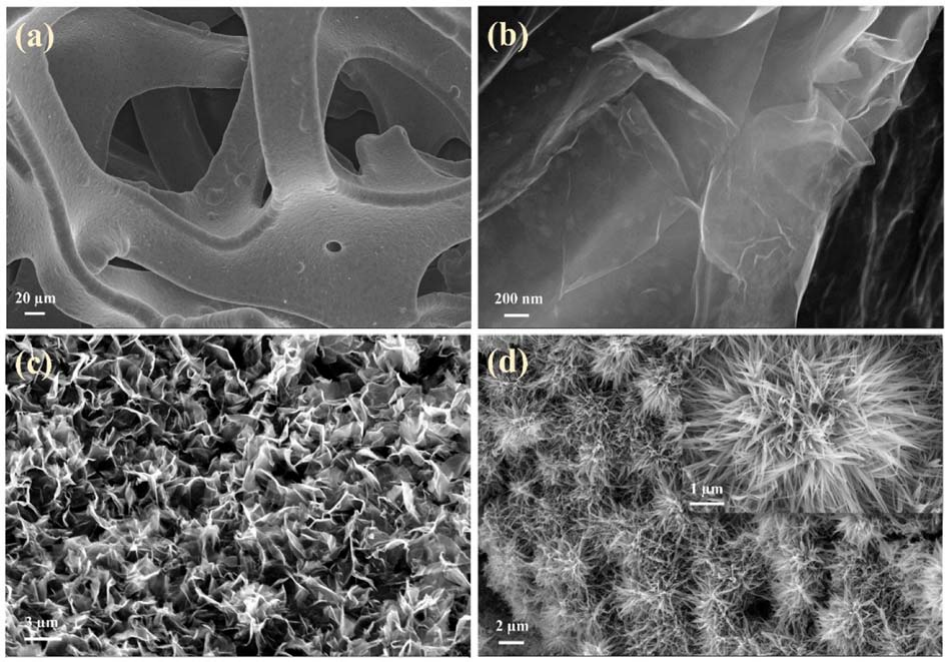
Morphological images of (a) bare NF, (b) rGO/NF, (c) SCN, and (d) SCGN; the inset in (d) is the plot of SCGN.

The morphologies and microstructures of the composite electrodes were examined through SEM, TEM, HRTEM, and SAED. According to Fig. 4a, the processed nickel foam (NF) exhibits a three-dimensional porous network structure with a smooth and flat surface. The surface of the rGO-loaded nickel foam is covered with a semi-transparent and wrinkled film, as depicted in Fig. 4b. The single metal oxide Co_3_O_4_, as illustrated in Fig. S1a and b,† grows in an elliptical sheet-like structure, overlapping on the nickel foam substrate while adding rGO forms uniformly dispersed nanoneedles. Similarly, the monometallic Sm_2_O_3_ stacked in layers on NF formed a large number of tightly packed nanoflakes, the addition of rGO introduces a more sparse and irregular lamellar structure, and rGO films with fold-like structures are also found at the edges of SGN (Fig. 4c and d). This result implies that the incorporation of rGO contributes to the dispersion of the metal oxides, thus increasing the specific surface area of the composites to some extent. Furthermore, the inclusion of rGO has altered the morphology of metal oxides towards a more favourable shape for electrolyte ion penetration. As shown in Fig. 4d, SCGN nanoparticles grow on the NF substrate in highly ordered dandelion-like clusters, with an average cluster size of 4 μm comprising numerous nanorods. These nanorods maintain a certain distance from each other, which proves advantageous in mitigating the volume expansion stemming from rapid, reversible electrochemical reactions. Compared to the tremella-like form of SGN in Fig. 4c, SCGN exhibits a larger specific surface area, further affirming rGO's capacity to augment the specific surface area.^64^ Overall, the dandelion-like cluster morphology resembles “tentacles” growing on the current collector, thereby increasing the contact area between the electrode and electrolyte, facilitating the transfer of ions and electrons, and providing more active sites to increase electrode conductivity. The element mapping analysis is demonstrated in Fig. S2,† which illustrates the uniform distribution of Sm, Co, C, and O elements. The findings provide evidence that rGO, Sm_2_O_3,_ and Co_3_O_4_ are evenly distributed in a floral structure on the NF, corroborating the SEM analysis. Notably, the highest proportion of elements is C, accounting for 36.98%, followed by O, Co, and Sm elements at 35.14%, 13.06%, and 2.11%, respectively (Fig. S3†).

**Fig. 5:**
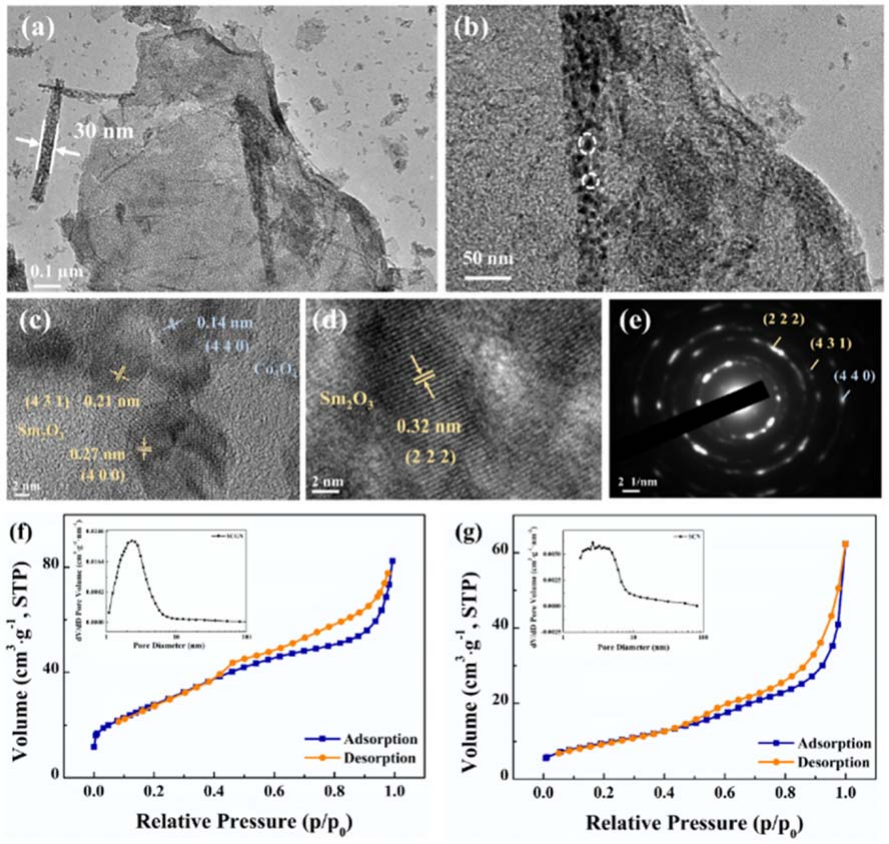
TEM images (a and b), HRTEM images (c and d), and the SAED pattern (e) of the SCGN; nitrogen adsorption and desorption isotherm of (f) SCGN and (g) SCN with the pore size distribution in the insets respectively.

To further analyse the dandelion-like structure of SCGN, TEM tests were performed. As illustrated in Fig. 5a, the distinctive flower rod configuration within the floral cluster is distinctly visible, with the rod's dimensions measuring approximately 30 nm. Surrounding the rod, a delicate and crinkled rGO thin film is discernible, thus validating the successful amalgamation of Sm_2_O_3_ and Co_3_O_4_ with rGO, and this result is also consistent with SEM. From Fig. 5b, it can be observed that the flower rod structure contains nanoparticles with highly porous. This intricate porous framework engenders an increased interface between the active material and the electrolyte solution, facilitating expedited pathways for the diffusion of ions and electrons and enabling rapid redox reactions during the charging and discharging process.^65^Fig. 5c and d shows HRTEM with a lattice spacing of 0.27 nm, 0.32 nm, and 0.21 nm corresponding to (440), (222), and (431) crystal planes of Sm_2_O_3_, respectively, and 0.14 nm lattice spacing corresponding to (440) crystal planes of Co_3_O_4_. Fig. 5e presents a selected area electron diffraction (SAED) pattern, and the actual distance from the diffraction spots to the centre of the transmitted beam can be calculated to determine the (222) and (431) crystal planes of Sm_2_O_3_, as well as the (440) crystal plane of Co_3_O_4_. The above results consistently align with the XRD, XPS, and Raman results, further proving the successful synthesis of SCGN.

Fig. 5f and g depict the nitrogen physical adsorption–desorption isotherms of SCGN and SCN. The inset graph shows the pore size distribution, which is evenly distributed and concentrated within the 2–5 nm range. The average pore sizes are measured to be 4.69 nm and 10.19 nm for SCGN and SCN, respectively, indicating that both materials possess a mesoporous structure.^66^ Further evaluation of the BET specific surface areas reveals values of 102.60 m^2^ g^−1^ and 34.68 m^2^ g^−1^ for SCGN and SCN, respectively. Evidently, SCGN possesses a substantially larger specific surface area, a characteristic that augments the abundance of active sites for enhanced interactions between the active species and electrolyte ions. It also demonstrates that the dandelion-like structure of SCGN promotes a faster diffusion rate, resulting in quicker electron transfer between the active substance and electrolyte.

### 3.3 Growth mechanism

Based on the above discussion, we have presented a possible growth step. The following process can be used to describe the decomposition of urea in an autoclave:
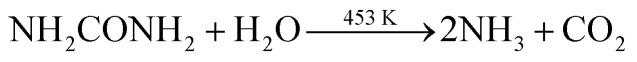

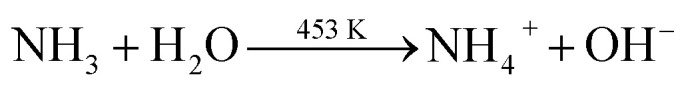

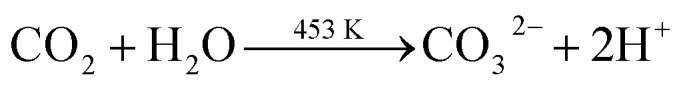


During the hydrothermal reaction, the solution gradually becomes alkaline with the release of NH_3_, favouring the heterogeneous nucleation of Co and Sm. At the initial stage of the hydrothermal reaction, Co^2+^, Sm^3+^, OH^−^ and CO_3_^2−^ in the aqueous solution are easily adsorbed by GO, which contains a large number of hydrophilic groups on the surface, and uniformly bind all the particles through the interaction forces including van der Waals forces and hydrogen bonds. The amorphous Co nuclei and amorphous Sm nuclei formed in the reaction medium gradually form nanoparticles at the active nucleation centre. The initially formed nanoparticles continuously aggregated and spontaneously grew into dandelion-like clusters consisting of several nanorods on the NF substrate with minimal surface energy. Meanwhile, carbon dioxide and water vapour were generated under annealing at 300 °C for 2 h, which contributed to the formation of porous nanostructures in the composites. The reactions of the hydrothermal and annealing processes were as follows:
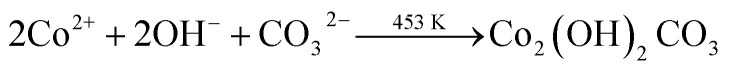

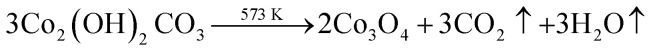

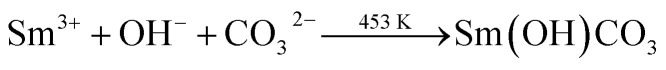

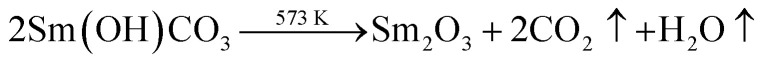


### 3.4 Electrochemical properties

#### 3.4.1 The electrochemical performance of Sm_2_O_3_/Co_3_O_4_/rGO/NF composite

**Fig. 6:**
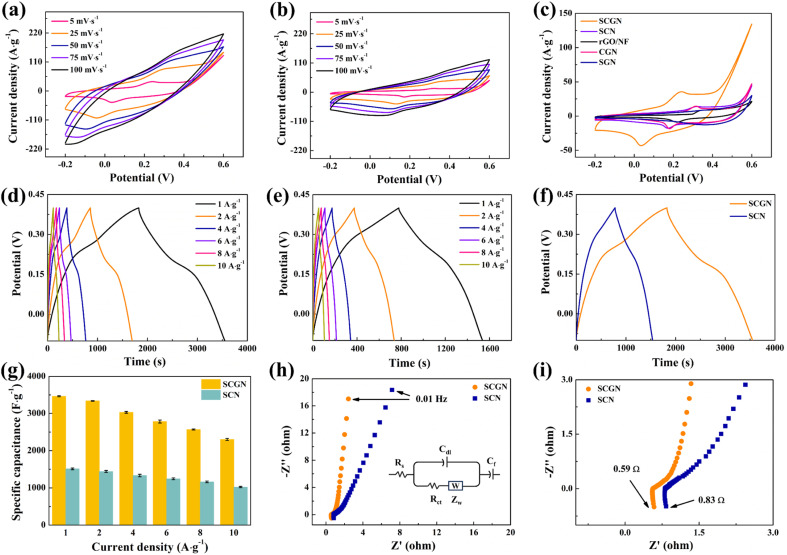
CV curves of (a) SCGN and (b) SCN electrode under different scan rates; (c) comparison CV curves of SCGN, SCN, rGO/NF, CGN, SGN; GCD curves of (d) SCGN and (e) SCN electrode at different current densities; (f) comparison GCD curves of SCGN and SCN electrode at 1 A g^−1^; (g) specific capacitances of SCGN and SCN electrode at different current densities; (h) the Nyquist plots of SCGN and SCN electrode; (i) high-frequency region of the Nyquist plots. All of the electrochemical measurements described above were obtained in 6 mol L^−1^ KOH solution.

To investigate the electrochemical performance of the prepared Sm_2_O_3_/Co_3_O_4_/rGO/NF composite (SCGN) electrode material, the CV, GCD, and EIS tests were conducted in a three-electrode system employing a 6 mol L^−1^ KOH electrolyte solution. The electrochemical process of SCGN and SCN electrodes at scanning rates of 5–100 mV s^−1^ is shown in Fig. 6a and b. The CV curve reveals distinct oxidation-reduction peaks for both SCGN and SCN electrode materials, indicating that the electrode's specific capacitance is primarily derived from pseudocapacitive behaviour, likely linked to the faradaic redox reactions of Sm–O/Sm–O–OH and Co–O/Co–O–OH.^67^ By comparing the CV curves of SCN electrode material at a scan rate of 5–100 mV s^−1^, it is observed that the oxidation and reduction peak currents of SCN are lower. This divergence underscores the dandelion-like porous structure's efficacy in facilitating ion entry from the electrolyte into the electrode interior, outperforming sheet-like configurations. Remarkably, even at an elevated scan rate of 100 mV s^−1^, observable oxidation and reduction peaks in the CV curve attest to the prompt kinetics of oxidation and reduction reactions, indicative of swift electron and KOH electrolyte ion exchanges within the composite electrode material. Furthermore, from Fig. 6a, it can be observed that as the scanning rate increases from 5 mV s^−1^ to 100 mV s^−1^, the integrated area of the CV curve decreases, indicating a gradual decrease in capacitance values. This suggests that the electrochemical capacitance process is influenced by concentration polarisation or diffusion-controlled electrochemistry. Additionally, the slight shift in oxidation and reduction peaks towards higher and lower potentials is attributed to the elevated diffusion resistance within the electrode material.^63^ Moreover, the shape of the CV curve remains unchanged, indicating excellent electrochemical reversibility of the electrode. The investigation of electronic and ionic conductivity explores the vital role of charge carriers in the embedding and extraction processes within electrode materials. The following mechanisms describe the electrochemical processes that occur in electrolyte solutions:Co_3_O_4_ + OH^−^ + H_2_O ↔ 3CoOOH + e^−^CoOOH + OH^−^ ↔ CoO_2_ + H_2_O + e^−^Sm_2_O_3_ + H_2_O ↔ 2SmOOHSmOOH + OH^−^ ↔ Sm(OH)_2_ + e^−^

Fig. 6c shows a comparison of cyclic voltammetry curves for SCGN, SCN, SGN, CGN, and rGO/NF at a scanning rate of 5 mV s^−1^. By comparing the curves, it is visually evident that the CV curve area of the SCGN composite electrode is significantly larger than that of rGO/NF and other single oxides, indicating that the SCGN composite electrode has superior specific capacitance performance. The amalgamation of Co and suitable Sm engenders excellent surface and redox processes. Coupled with rGO, which boasts a larger specific surface area, it augments active sites available for Sm_2_O_3_ and Co_3_O_4_ loading. Consequently, it expedites faradaic redox reactions of Sm_2_O_3_ and Co_3_O_4_, bolstering electronic conductivity and electrochemical capacity. In addition, the Sm_2_O_3_ material has higher oxygen diffusion and increased charge carrier concentration, enhancing electronic and ionic conductivity. The SCGN electrode material induces metallic behaviour, thereby refining conductivity. The composition of the same metal family reduces the thermal expansion coefficient. In contrast, the synergy between two different groups, especially transition metals and rare earth metals, increases oxygen vacancies caused by charge compensation.

Following that, the charge–discharge test was conducted using a constant current method. The test was performed in a three-electrode system with a voltage range of −0.6 V to 0.6 V. Fig. 6d illustrates the electrochemical performance and specific capacitance of the composite electrode at current densities of 1–10 A g^−1^. It can be seen that the SCGN electrode displays an apparent plateau in both charge and discharge, and the non-linear GCD curve further confirms the pseudo-capacitance behaviour of the SCGN. As the current density decreases, the charging and discharging times become longer. In addition, the symmetrical GCD curve shape indicates an excellent reversible redox reaction occurring. The specific capacitance at different current densities is calculated from eqn (1). The bare nickel foam shows a meagre specific capacity value of less than 1.5 F g^−1^, which was therefore ignored when calculating the specific capacity at various current densities. The maximum specific capacitance reaches 3448 F g^−1^ at a current density of 1 A g^−1^. At current densities of 2, 4, 6, 8, and 10 A g^−1^, the specific capacitance corresponds to 3346.4, 3028, 2794.8, 2571.2, and 2292 F g^−1^, respectively. The calculated results show that the specific capacitance of SCGN decreases with increasing current density mainly because only the active material on the surface of the electrode can react completely at higher current densities. The lower ion diffusion rate often fails to meet the high redox reaction rate required at high current densities, resulting in a gradual decrease in specific capacitance. The GCD curve in Fig. 6e represents the performance of SCN electrode material. The specific capacitance of the SCN electrode material is 1515.8, 1446, 1341.6, 1248, 1166.4, and 1024 F g^−1^, respectively, for the same current density and potential window according to eqn (2). Fig. 6f displays the comparative GCD curves for SCGN and SCN at a current density of 1 A g^−1^. It can be observed that the charge and discharge times of SCGN are significantly prolonged due to the addition of rGO, which is consistent with the increased specific capacitance. The specific capacitance of SCGN is approximately 2.3 times that of SCN (1515.8 F g^−1^). As shown in Fig. 6g, the prepared composite electrode exhibits the highest capacity among the other prepared electrodes. The results for specific capacitance also indicate that the dandelion-like structure of SCGN facilitates rapid ion access to the electrode and supports redox reactions with high specific capacitance. This further illustrates the role of rGO in optimising the electrode, enhancing capacitance, and improving electrochemical performance.

Furthermore, internal resistance is one of the intrinsic factors affecting the electrochemical performance of electrode materials. EIS testing was carried out in the open-circuit voltage range of 100 kHz to 0.01 Hz, as shown in Fig. 6h. Fig. 6i depicts the intercept of the curve with the *X*-axis in the high-frequency region, representing the internal resistance (*R*_s_), which is influenced by the ion resistance of KOH electrolyte, rGO, Co_3_O_4_, and Sm_2_O_3_, as well as the contact resistance at the electrolyte interface. Comparing SCGN with SCN, it is shown that neither SCGN nor SCN shows a precise semicircular shape in the high-frequency region, indicating a low charge transfer resistance (*R*_ct_) at the electrode–electrolyte interface. The slope in the low-frequency region is an essential parameter for measuring the Warburg impedance caused by proton diffusion. By comparison, SCGN has a more significant slope in the low-frequency region, suggesting a more negligible ion diffusion resistance. The smaller *R*_s_ and *R*_ct_ of SCGN once again effectively confirm that the addition of rGO enhances the inherent electronic conductivity and electrochemical activity of the composite material.

**Fig. 7:**
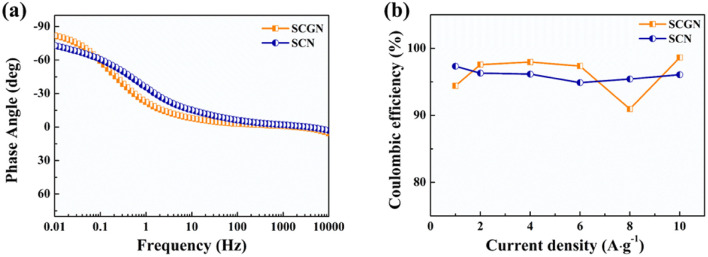
(a) Phase angle *vs.* frequency analysis of SCGN and SCN electrode; (b) the coulombic efficiencies of SCGN and SCN electrode at different current densities.

Fig. 7a illustrates the phase angle–frequency curves for the SCGN and SCN electrode materials. Improved capacitive behaviour is indicated by a phase angle closer to 90°. From the curve, it is evident that at 0.01 Hz, the phase angle of SCGN is 81.9°, higher than the phase angle of SCN (73.1°). The rapid decrease in phase angle within the range of 0.01–10 Hz indicates rapid electrolyte ion penetration into the electrode's interior, which can be attributed to the dandelion-like flower structure of SCGN. Additionally, the phase angle curve of SCGN remains stable under applied frequencies and shows almost no variation within the range of 10–1000 Hz, suggesting that this composite electrode material exhibits stable capacitive performance in this frequency range.

Coulombic efficiency serves as an indicator of electrode material reversibility and can help predict the “lifetime” of the material. The coulombic efficiency is determined by eqn (4):4
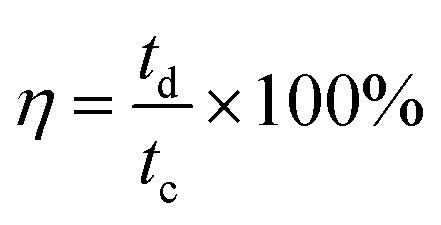
where *η* is the Coulomb efficiency, *t*_d_ is the discharge time, and *t*_c_ is the charging time.

The coulombic efficiency graph of SCGN electrodes at different current densities is illustrated in Fig. 7b. The coulombic efficiency of SCGN electrode material is approximately 97.3%, indicating excellent reversibility as a pseudocapacitive material. The above analysis suggests that SCGN is a promising electrode material for supercapacitors.

**Fig. 8:**
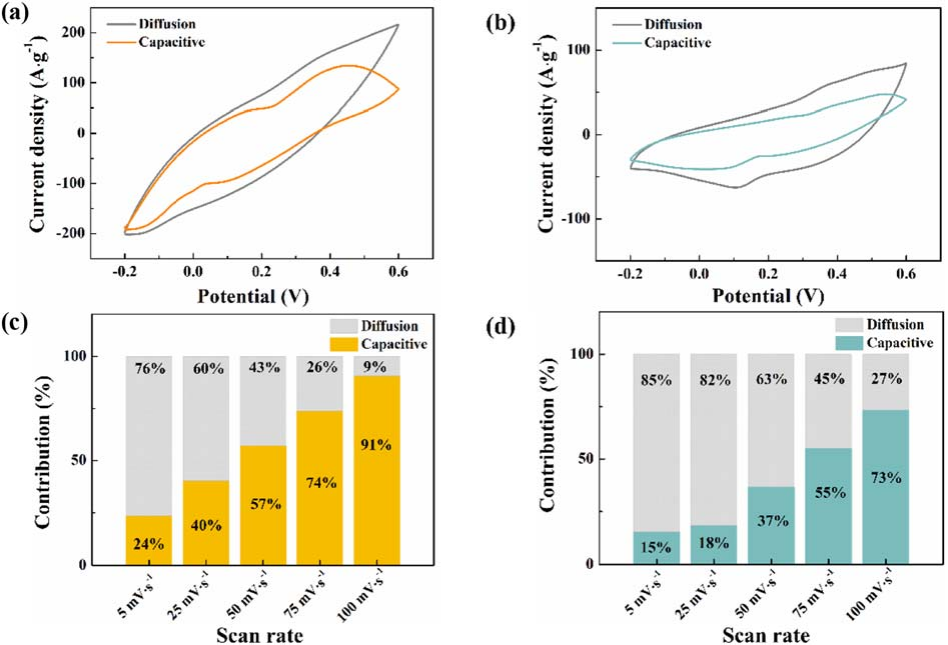
Comparison of the capacitive contribution and the diffusion-controlled contribution fraction between (a and c) SCGN and (b and d) SCN.

To further understand the supercapacitor storage behaviour of Sm_2_O_3_/Co_3_O_4_/rGO/NF composite (SCGN) and Sm_2_O_3_/Co_3_O_4_/NF composite (SCN), a kinetics study based on CV curves (Fig. 8a and b) was performed using the following equation:^45^*i* = *ab*^*v*^where *i* is the current, *ν* is the scan rate, *a* and *b* are adjustable values. A value of 0.5 for *b* indicates a diffusion-controlled process due to cation intercalation, whereas a value of 1 indicates capacitive behaviour controlled by surface Faraday redox reactions.^68^ On this basis, the total current is divided into capacitive (*k*_1_*v*) and diffusion control (*k*_2_*v*^1/2^) components with the following relationship:*i*(*V*) = *k*_1_*v* + *k*_2_*v*^1/2^where *i* is the current at a fixed voltage (*V*), *k*_1_ and *k*_2_ are constants.

The contribution between these two different processes was tested at different scan rates. Fig. 8c and d give the corresponding capacitance contributions at different scan rates. The capacitance capacity gradually increases as the scan rate increases, and finally reaches a maximum value at 100 mV s^−1^. It is noteworthy that the overall contribution of SCGN is higher than that of SCN at any scan rate. The results of the capacitive contribution show that the capacitive charge storage of SCGN accounts for more than 90% of the total capacity, which further proves that it has a high multiplicity. Thus, the corresponding Faraday reaction is not a kinetically controlled process but a fully diffusion-limited one.

#### 3.4.2 SCGN//SCGN symmetric supercapacitor

**Fig. 9:**
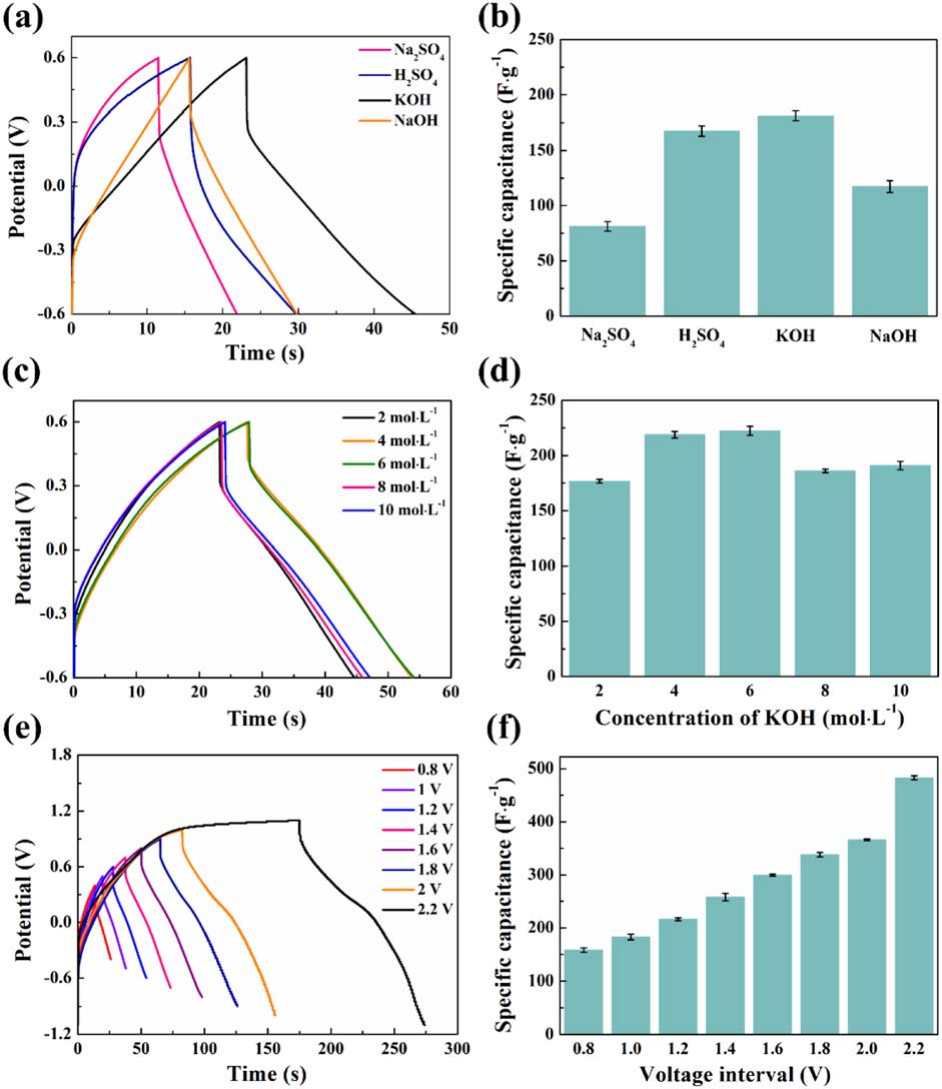
(a) GCD curves of SCGN electrode for different types of electrolytes at 10 A g^−1^; (b) specific capacitances of SCGN electrode for different types of electrolytes at 10 A g^−1^; (c) GCD curves of SCGN for different molar concentrations of KOH at 10 A g^−1^; (d) specific capacitances of SCGN electrode for different molar concentrations of KOH at 10 A g^−1^; (e) GCD curves of SCGN electrodes at different voltage windows at 10 A g^−1^; (f) specific capacitances of SCGN electrodes at different voltage windows at 10 A g^−1^.

To assess the impact of different types of electrolyte solutions on the specific capacitance of supercapacitors, SCGN//SCGN supercapacitors were tested in 1 mol L^−1^ KOH and compared with the corresponding specific capacitance of 1 mol L^−1^ H_2_SO_4_ electrolyte, 1 mol L^−1^ NaOH electrolyte, and 1 mol L^−1^ Na_2_SO_4_ electrolyte. The electrochemical performance comparison of several electrolytes is shown in Fig. 9a and b. The research found that SCGN//SCGN supercapacitors exhibit better electrochemical performance in KOH (specific capacitance of 185.8 F g^−1^). Due to the fact that Sm_2_O_3_ and CO_3_O_4_ are both oxides, acidic electrolyte (H_2_SO_4_) will to some extent damage the electrode structure, resulting in lower specific capacitance of SCGN//SCGN supercapacitor in H_2_SO_4_ electrolyte compared to KOH electrolyte. The neutral electrolyte is more beneficial and less corrosive in terms of the safety of the energy storage device. However, it exhibits the worst electrochemical performance, which can be explained by ionic radius, ionic hydration shell radius, ion conductivity, and ion migration in the electrolyte. The size of the Na^+^ hydrated ion is 3.58 Å, with an ionic conductivity of 50.11 S cm^2^ mol^−1^, while the size of the K^+^ hydrated ion is 3.31 Å, with an ionic conductivity of 73.5 S cm^2^ mol^−1^. The hydrated negative anion radius is OH^−^ (3.00 Å) < SO_4_^2−^ (5.33 Å).^69^ The mesoporous structure of SCGN composite materials can easily accommodate small-sized K^+^ ions and adsorb charged hydroxide anions (OH^−^) negatively. Considering its superior conductivity and ionic migration, the KOH electrolyte is expected to offer optimal electrochemical performance.

Fig. 9c shows the typical GCD curves of SCGN//SCGN supercapacitors with different concentrations of KOH (2–10 mol L^−1^) in the voltage range of −0.6–0.6 V at a current density of 10 A g^−1^ and the corresponding specific capacitances are shown in Fig. 9d. The specific capacitance of the SCGN//SCGN supercapacitor increases with the increase of KOH concentration (the specific capacitance value increases from 178.3 F g^−1^ to 218.3 F g^−1^ as the KOH concentration increases from 2 mol L^−1^ to 6 mol L^−1^), reaching a maximum value at a KOH concentration of 6 mol L^−1^. The specific capacitance value decreases with a further increase in the concentration of the KOH electrolyte (when the KOH concentration is 10 mol L^−1^, the specific capacitance decreases to 191.7 F g^−1^). It is well known that the specific conductivity of the electrolyte is a key parameter determining its electrochemical performance. The specific conductivities corresponding to KOH concentrations of 2 mol L^−1^, 4 mol L^−1^, 6 mol L^−1^, 8 mol L^−1^, and 10 mol L^−1^ are 0.35 S cm^−1^, 0.53 S cm^−1^, 0.57 S cm^−1^, 0.54 S cm^−1^, and 0.47 S cm^−1^, respectively.^70^ Therefore, in the electrolyte solution environment of 6 mol L^−1^ KOH, the SCGN//SCGN supercapacitor exhibits optimal electrochemical performance.

**Fig. 10:**
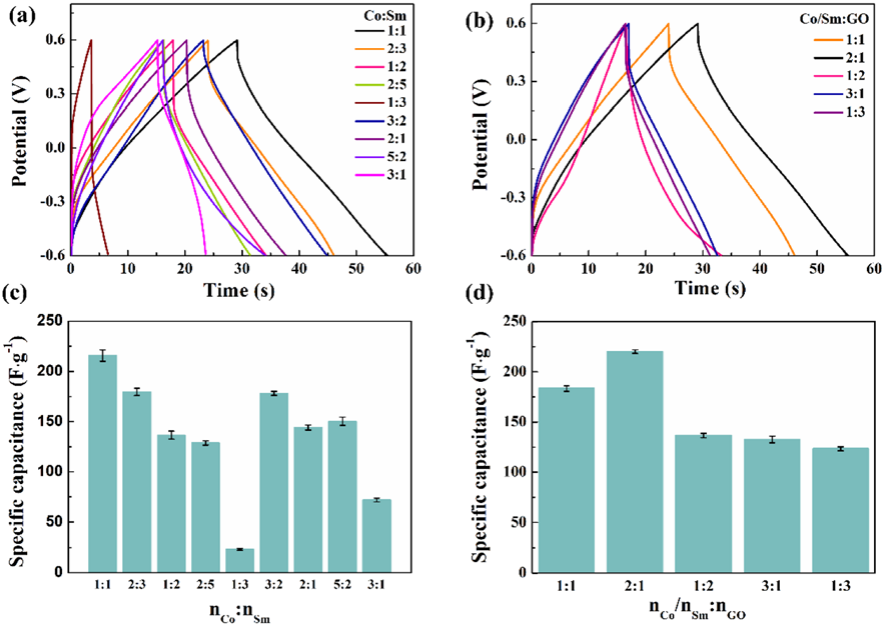
(a and b) GCD curves and (c and d) specific capacitances of composite electrode materials prepared with different ratios of Co-sourced compounds, Sm-sourced compounds and GO at 10 A g^−1^.

**Fig. 11:**
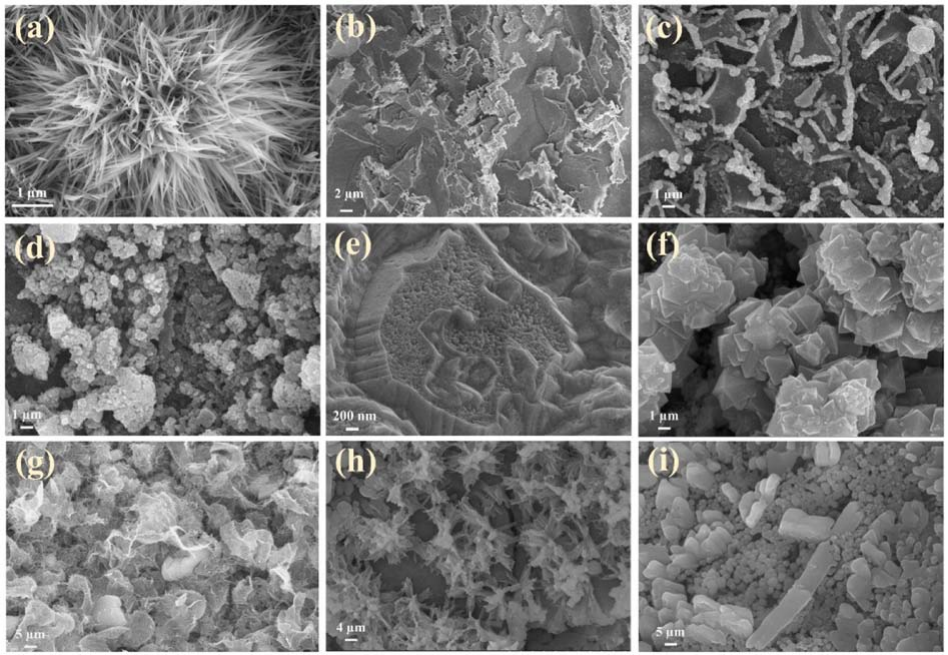
Morphological images of composite electrode materials were prepared with different ratios of Co-sourced compounds and Sm-sourced compounds with (a) *n*_Co_ : *n*_Sm_ = 1 : 1; (b) *n*_Co_ : *n*_Sm_ = 2 : 3; (c) *n*_Co_ : *n*_Sm_ = 1 : 2; (d) *n*_Co_ : *n*_Sm_ = 2 : 5; (e) *n*_Co_ : *n*_Sm_ = 1 : 3; (f) *n*_Co_ : *n*_Sm_ = 3 : 2; (g) *n*_Co_ : *n*_Sm_ = 2 : 1; (h) *n*_Co_ : *n*_Sm_ = 5 : 2; (i) *n*_Co_ : *n*_Sm_ = 3 : 1.

**Fig. 12:**
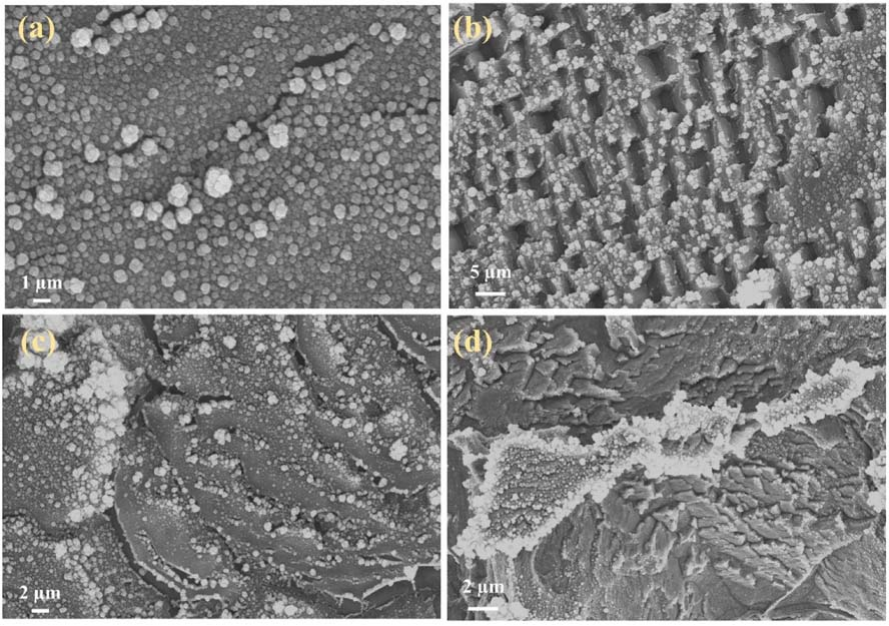
Morphological images of composite electrode materials were prepared with different ratios of Co-sourced compounds/Sm-sourced compounds and GO with (a) *n*_Co_/*n*_Sm_ : *n*_GO_ = 1 : 1; (b) *n*_Co_/*n*_Sm_ : *n*_GO_ = 1 : 2; (c) *n*_Co_/*n*_Sm_ : *n*_GO_ = 3 : 1; (d) *n*_Co_/*n*_Sm_ : *n*_GO_ = 1 : 3.

The composite electrode materials were prepared with different ratios of Co-sourced compounds, Sm-sourced compounds and GO, and assembled into symmetric supercapacitors. Fig. 10a shows the typical GCD curves with different ratios of Co-sourced and Sm-sourced compounds in the voltage range of −0.6 to 0.6 V at a current density of 10 A g^−1^. The corresponding specific capacitances are shown in Fig. 10c. From the comparative GCD curves, it can be seen that the longest charging and discharging time and the largest specific capacitance value (220 F g^−1^) are obtained when the molar ratio of Co-sourced compounds and Sm-sourced compounds is 1 : 1. The structure of the electrode material is a crucial factor that affects the speed of ion transport and the size of the active surface area, so SEM of the composites with different ratios was determined. As can be seen from the SEM images (Fig. 11), the electrode materials prepared with other molar ratios of Co-sourced and Sm-sourced compounds form inhomogeneous nanoparticles, all showing varying degrees of large-scale aggregation. As the ratio difference between *n*_Co_ and *n*_Sm_ increased, the particle stacking phenomenon is gradually apparent. Among them, the complexes prepared with *n*_Co_ : *n*_Sm_ = 1 : 3 and *n*_Co_ : *n*_Sm_ = 3 : 1 grow a large number of nanorods or nanosheets on the nickel foam base, which almost crowd the reticular voids of the nickel foam base. The electrochemical properties of the electrode materials prepared with different molar ratio values of Sm/Co and GO were investigated under the same test conditions. Fig. 10b demonstrates its GCD curves, and Fig. 10d compares specific capacitance. The specific capacitance is maximum when *n*_Co_/*n*_Sm_ : *n*_GO_ = 2 : 1. Fig. 12a–d shows that Sm_2_O_3_ and Co_3_O_4_ nanoparticles grow less and unevenly when too much GO is added, and there is a collapse of the structure, which is unfavourable to the growth of Sm_2_O_3_ and Co_3_O_4_ nanoparticles; whereas, too little GO is easy to appear the phenomenon of tight stacking. All of the above structures are unfavourable for electrolyte entry, and the blocked ion transport channel will affect the ion transport and the mass transfer ability of electrolytes in the electrode, thus limiting the electrochemical performance of the materials. Experimental results show that the most suitable active material ratio should be 2 : 2 : 1 to obtain the best overall electrochemical performance (SEM is shown in Fig. 11a).

Fig. 9e and f show the GCD curves and specific capacitance comparison of SCGN//SCGN supercapacitors at different voltage windows when the current density is 10 A g^−1^, respectively. As the voltage window increases, the discharge time of the supercapacitor gradually increases, and the corresponding value of the specific capacitance rises accordingly. Although a larger voltage window helps to increase the specific capacitance and energy density of supercapacitors, the physicochemical aspects involving electrolyte stability and the water decomposition process cannot be ignored. For symmetric supercapacitors, Δ*E*_1_ = −Δ*E*_2_ and *ω*^*β*^ = *ω*^*α*^ (where *ω*^*β*^ and *ω*^*α*^ are the electrochemical potentials of the positive and negative electrodes, respectively, and Δ*E*_1_ and Δ*E*_2_ are the surface dipole potentials of the positive and negative electrodes, respectively), the additional potential window of a symmetric supercapacitor becomes zero.^71^ Therefore, the operating voltage is determined by the dissociation energy of the electrolyte, which can be up to 1.23 V when an aqueous electrolyte is used. Supercapacitors generally have a voltage window lower than 1.2 V to avoid water decomposition (decomposition tension of 1.23 V).^72^ A voltage window that is too high tends to cause irreversible reactions to occur, which affects the cycling stability of the supercapacitor. Therefore, we chose a stabilising potential window of 1.2 V to enable the prepared SCGN electrodes to maintain high electrochemical performance for extended periods.

**Fig. 13:**
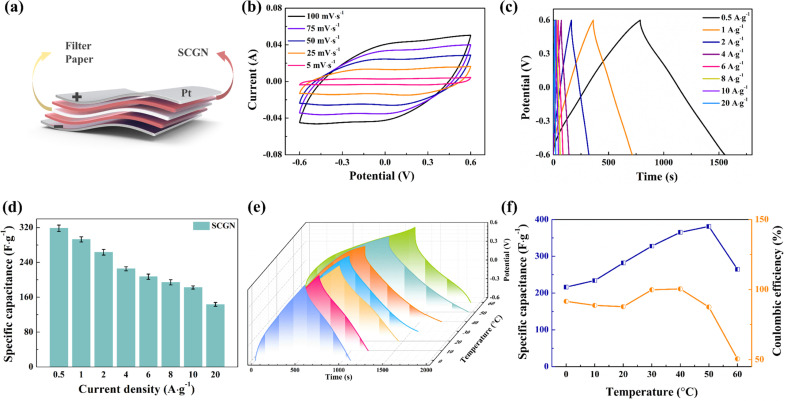
(a) 3D schematic diagram of the SCGN//SCGN symmetric supercapacitor device; (b) CV curves of the symmetric supercapacitor under different scan rates; (c) GCD curves of the supercapacitor at different current densities; (d) specific capacitances of the device at different current densities; (e) GCD curves of the supercapacitor at different temperatures; (f) specific capacitances and coulombic efficiencies of the supercapacitor at different temperatures.

To further assess the practical performance of the composite electrode materials, two SCGNs were employed as the anode and cathode, while filter paper served as the septum to construct the SCGN//SCGN supercapacitor (SSC), as illustrated in Fig. 13a. A series of electrochemical tests such as CV, GCD, and EIS were carried out in 6 mol L^−1^ KOH solution to investigate the electrochemical performance of the supercapacitor. Fig. 13b shows the cyclic voltammetric curves (CV) under different scan rates (5–100 mV s^−1^, −0.6 to 0.6 V voltage range). At a scan rate of 5 mV s^−1^, the CV curve manifests a near-rectangular mirror shape, indicating its exceptional capacitive behaviour. Even at scan rates of up to 100 mV s^−1^, the CV curve remains well in the near-rectangular shape, showing the excellent stability and multiplicative properties of the simple SSC, which can be attributed to the porous structure of the electrode material, allowing efficient transport of electrolyte ions and shortening the diffusion distance to the internal surface.

In addition, the charge and discharge curves at different current densities showed symmetrical linear curves in Fig. 13c, indicating that the SCGN//SCGN symmetrical supercapacitor has good capacitive performance. The favourable capacitive behaviour of the electrode material stems from several key attributes: (1) the good electrical conductivity of rGO, which mitigates the internal resistance of Sm_2_O_3_–Co_3_O_4_ particles; (2) the homogeneous distribution of Sm_2_O_3_–Co_3_O_4_ particles on the surface of rGO promotes its charge conduction, which allows a fast redox reaction to occur. The specific capacitance values for different current densities were obtained from eqn (1) for this capacitor, and the results are shown in Fig. 13d. The specific capacitance corresponds to 319.75, 294.83, 264.33, 230, 209.5, 195.33, 183.33, and 143.33 F g^−1^ for current densities of 0.5, 1, 2, 4, 6, 8, 10, and 20 A g^−1^, respectively.

Temperature stands as a crucial parameter that determines the operational lifespan, safety, and thermal behaviour of supercapacitors in practical environments. As shown in Fig. 13e, the GCD curve of the SCGN//SCGN device retains symmetrically triangular within the temperature range of 0–60 °C. The calculated results concerning specific capacitance and coulombic efficiency at different temperatures are shown in Fig. 13f. At a temperature of 50 °C, the specific capacitance reaches its maximum value of 381.25 F g^−1^. A decline in specific capacitance accompanies lower temperatures. At lower temperatures, the high freezing point of the aqueous electrolyte hampers ionic conductivity, while limited compatibility between electrode material and electrolyte augments resistance and decelerates reaction rates. Conversely, excessively high temperatures may jeopardise the porous structure of the SCGN material, impeding ion detachment from crystals and moderately diminishing ion diffusion rates, culminating in irreversible electrochemical processes. Therefore, at higher temperatures, coulombic efficiency undergoes a sharp downturn. Nevertheless, owing to the carbon-based electrode material (rGO) having a large surface area, moderate conductivity, adjustable porosity, and suitable surface charge storage mechanism, the SCGN//SCGN device still exhibits a specific capacitance of 264.58 F g^−1^ even at a temperature as high as 60 °C. Moreover, at 0 °C, the specific capacitance remains 216.29 F g^−1^. Overall, the performance of the SSC exhibits remarkable stability across the expansive operational temperature range of 0–60 °C.

**Fig. 14:**
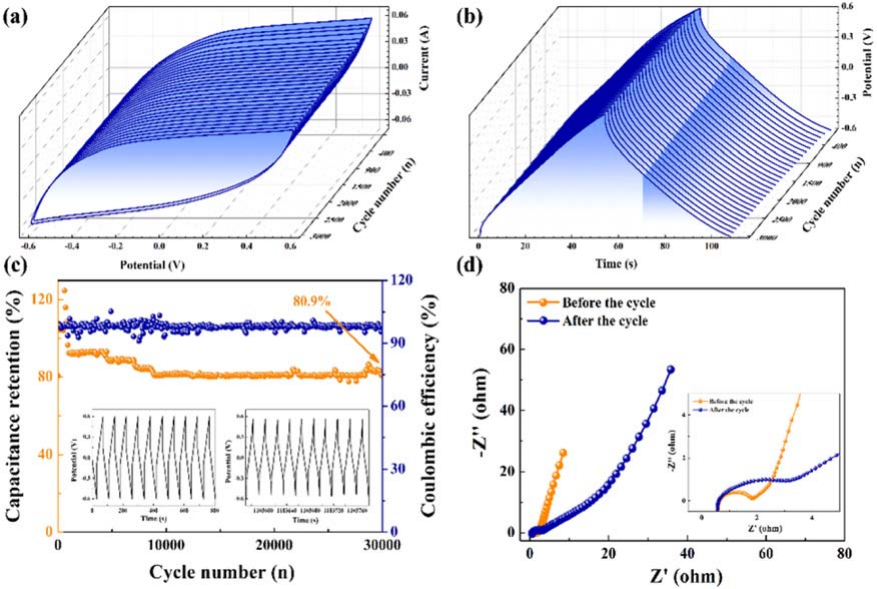
Changes in the (a) 3D-CV curves and (b) 3D-GCD curves as a function of a number of cycles for SCGN//SCGN at 200 mV s^−1^; (c) the capacitance retention and coulombic efficiency at a current density of 10 A g^−1^ for 30 000 cycles (inset: the GCD curve for the first 10 cycles and the last 10 cycles out of 30 000 cycles); (d) the Nyquist plot of the symmetric supercapacitor before and after cycles with the high-frequency region of the Nyquist plots in the inset.

As shown in Fig. S4,† a constant current charge–discharge cycle technology was employed to perform 3000 charge–discharge tests at a current density of 10 A g^−1^ within the potential window range of −0.6 to 0.6 V. The specific capacitance exhibited incremental growth during the initial 600 cycles, potentially attributed to the gradual activation of active sites within the electrode during the persistent charge–discharge processes. Following 600 cycles, the specific capacitance gradually decreased until reaching a capacity retention of 93.2% at 3000 cycles. Fig. 14a and b illustrates three-dimensional (3D) CVs and GCDs obtained at a scan rate of 100 mV s^−1^. In these three-dimensional plots, the variation of CVs and GCDs with the number of cycles is more visible, and the CV and GCD curves maintain a good shape. The stability of the electrode stands as a pivotal determinant in the successful application of supercapacitors. Fig. 14c shows the variation of capacity retention after 30 000 cycles of repeated charge–discharge at the same current density. Impressively, even after this extended cycling, the specific capacitance of the SSC device sustains above 80.9%. The shape of the Nyquist curve remains similar before and after cycling, and the equivalent circuit diagram remains unaltered. The *R*_ct_ value in the high-frequency region increases slightly, the slope of the straight line in the low-frequency region decreases, and the Warburg impedance increases (Fig. 14d). Overall, SCGN//SCGN supercapacitors demonstrate excellent cycling stability and possess practical long-term cycling capability as electrode materials for supercapacitors.

**Fig. 15:**
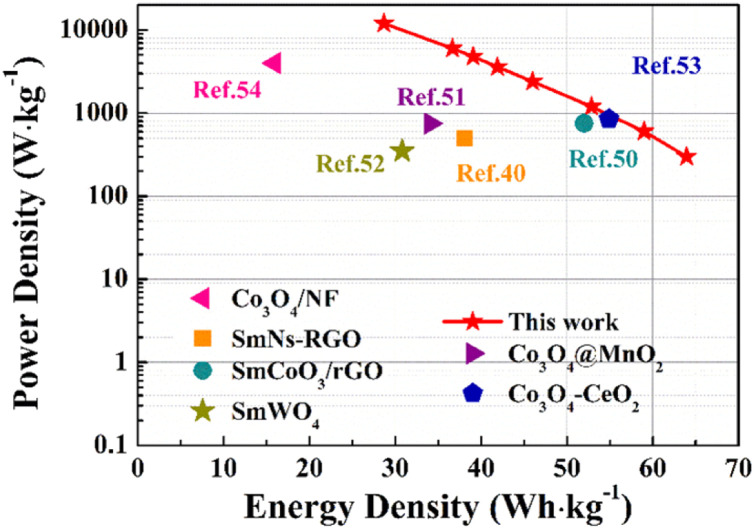
The Ragone plot of the device assembled using SCGN compared with the recently reported values in the literature.

As depicted in the Ragone diagram (Fig. 15), the prepared SCGN//SCGN devices exhibit noteworthy power density, reaching a maximum of 12 000 W kg^−1^ (accompanied by an energy density of 28.7 W h kg^−1^) and a maximum energy density of 64 W h kg^−1^ (with a power density of 300 W kg^−1^). Both of which are considerably higher than those of the previously reported supercapacitors.^58,73–77^ The electrochemical performance of the prepared composite electrode and previous relevant reports can be found in Table S1.† This indicates that the prepared SCGN electrode material performs excellently in various aspects, particularly its capacity characteristics and stability. Therefore, the high power density achieved by the SCGN//SCGN devices presents a promising outlook for their effective application in real-world scenarios.

## 4. Conclusions

A composite electrode featuring a dandelion-like structure (SCGN) consisting of Sm_2_O_3_, Co_3_O_4_, and 2D reduced graphene oxide was successfully synthesised onto a nickel foam substrate using a one-step hydrothermal method. The results show that the addition of Sm_2_O_3_ significantly improves the stability of the electrodes, and the double oxides Sm_2_O_3_ and Co_3_O_4_ effectively prevent the structure from collapsing during charging and discharging. In contrast, rGO can act as an electron transfer channel and provide good electrical contact for the dandelion-like flower-ball structure, which is able to provide high capacitance. By leveraging the synergistic effects of Sm_2_O_3_, Co_3_O_4,_ and reduced graphene oxide, the binder-free supercapacitor has achieved an ultrahigh specific capacitance of 3448 F g^−1^ at a current density of 1 A g^−1^. Notably, the SCGN electrode material exhibits an extensive operating temperature range (0–60 °C). At a temperature as high as 60 °C, the specific capacitance is 368.3 F g^−1^, and even at temperatures below 0 °C, the specific capacitance can still be maintained at 216.29 F g^−1^. The unique three-dimensional dandelion-like structure promotes ion diffusion and prevents disintegration of the structure during charging and discharging. Furthermore, the SCGN//SCGN device shows great potential for practical applications, with a specific capacitance of 319.75 F g^−1^ at 0.5 A g^−1^, a high energy density of 63.95 W h kg^−1^ at a power density of 300 W kg^−1^, which remains above 80.9% after 30 000 consecutive charge/discharge cycles. This work paves the way for using rare earth metals to enhance the stability of transition metals and potentially serves as electrode material for energy storage supercapacitors.

## Conflicts of interest

There are no conflicts to declare.

## Supplementary Material

RA-014-D3RA06352F-s001
