# Supplementary material for: Application of dandelion-like Sm2O3/Co3O4/rGO in high performance supercapacitors
Source: RSC Adv. 2024 Jan 9;14(3):2088–101. doi: 10.1039/d3ra06352f (PMC10775768; doi:10.1039/d3ra06352f)
Supplement: RA-014-D3RA06352F-s001 [file RA-014-D3RA06352F-s001.pdf]

Electronic Supplementary Material (ESI) for RSC Advances.
This journal is © The Royal Society of Chemistry 2024

Supporting Information

Application of dandelion-like Sm_2O_3/Co_3O_4/rGO in high performance supercapacitors

S1: Synthesis of Sm_2O_3/Co_3O_4/NF composite electrode material

0.75 mmol (0.333 g) of $Sm(NO_3)_3 \cdot 6H_2O$, 0.75 mmol (0.218 g) of $Co(NO_3)_2 \cdot 6H_2O$, and 1 mmol (0.060 g) of urea were added to 15 mL of distilled water and stirred vigorously with a magnetic stirrer for 1 h. The mixture was then transferred to a 25 mL tetrafluoroethylene lined autoclave containing a clean nickel foam (NF), and the reaction was carried out at a high temperature of 180 °C for 9 h. After completion of the reaction, the autoclave cooled to room temperature. The Sm_2O_3/Co_3O_4/NF precursor was repeatedly washed with deionized water and ethanol. The washed precursor was then dried at 60 °C for 8 h in an oven. Finally, the solid was annealed at 300 °C for 2 h in a muffle furnace to obtain the Sm_2O_3/Co_3O_4/NF composite electrode material.

S2: Synthesis of Co_3O_4/rGO/NF composite electrode material

Dissolve 0.75 mmol of $Co(NO_3)_2 \cdot 6H_2O$ and 1 mmol of urea in 7.5 mL of deionized water. Stir the mixture using a magnetic stirrer for 1 h to form a homogeneous solution. Then, 7.5 mL of a graphene oxide suspension with a concentration of 2 $mg \cdot mL^{-1}$ was added to the solution, followed by stirring for an additional 1 h. The treated NF and the mixed solution were then placed in a polytetrafluoroethylene high-pressure reactor and reacted at 180 °C for 9 h in an oven. The precursor was washed with ethanol and deionized water, followed by drying at 60 °C for 8 h. Finally, anneal the sample in a muffle furnace at 300 °C for 2 h to produce the Co_3O_4/rGO/NF composite electrode material.

S3: Synthesis of Sm_2O_3/rGO/NF composite electrode material

A homogeneous solution was obtained by dissolving 0.75 mmol $Sm(NO_3)_3 \cdot 6H_2O$ and 1 mmol urea in 7.5 ml distilled water and stirring magnetically for 1 h. The graphene solution (7.5 mL, 2 $mg \cdot mL^{-1}$) was mixed with the above solution and stirred

vigorously for 1 h. The cleaned NF was transferred with the solution into a 25 mL tetrafluoroethylene-lined autoclave, heated at 180 °C for 9 h, and then cooled naturally to room temperature. The Sm_2O_3/rGO/NF precursor was then washed several times with ethanol and distilled water and dried at 60 °C for 8 h. The Sm_2O_3/rGO/NF precursor was then annealed in a muffle furnace at 300 °C for 2 h to obtain the Sm_2O_3/rGO/NF composite electrode material.

S4: Synthesis of rGO/NF composite electrode material

0.75 mmol of $Sm(NO_3)_3{\cdot}6H_2O$ and 1 mmol of urea were mixed in 7.5 ml of distilled water. The solution and the cleaned nickel foam were then transferred to a 25 mL tetrafluoroethylene-lined autoclave and heated at 180 °C for 9 h. The rGO/NF precursor was washed with ethanol and deionized water and then dried in an oven at 60 °C for 8 h. Finally, it was annealed at 300 °C for 2 h in a muffle furnace to obtain the rGO/NF composite electrode material.

Results and discussion

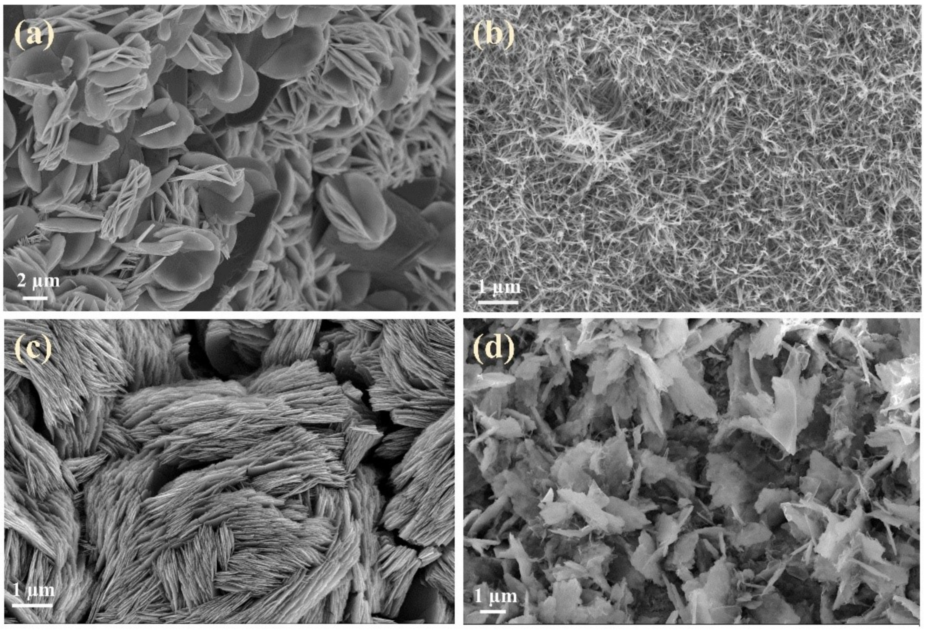

Fig. S1 The SEM images of various materials: (a) Co_3O_4/NF, (b) CGN, (c) Sm_2O_3/NF, and (d) SCN

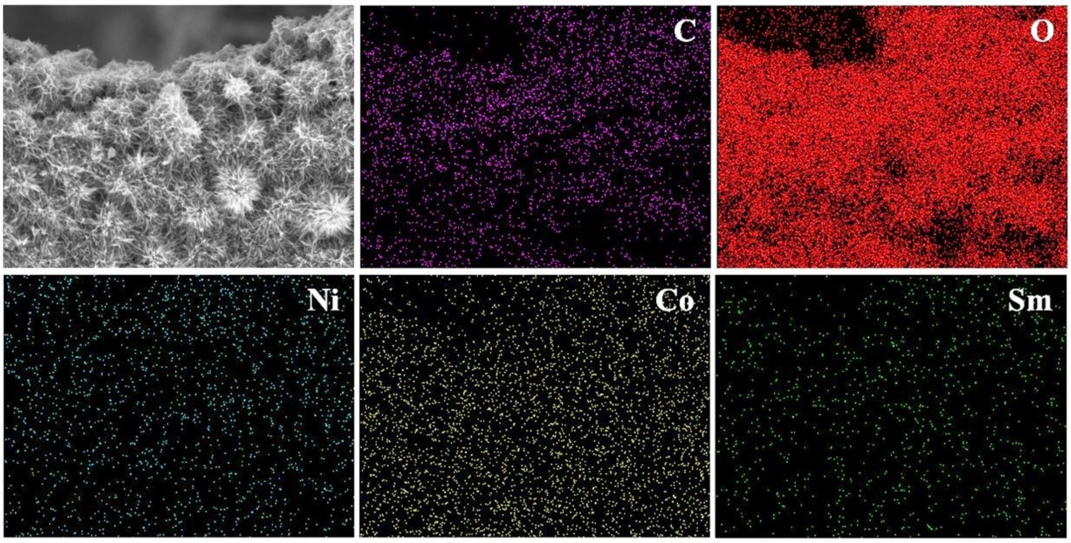

Fig. S2 EDS elemental mapping images of the composite (C, O, Ni, Co, and Sm)

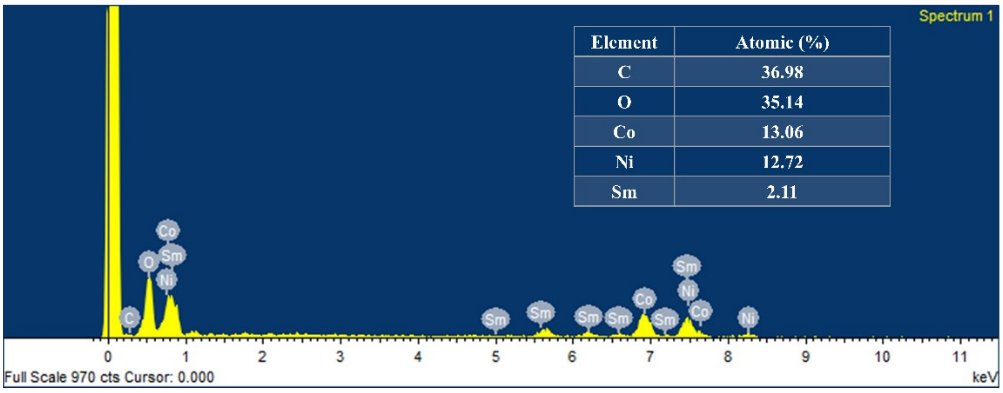

Fig. S3 EDS of SCGN

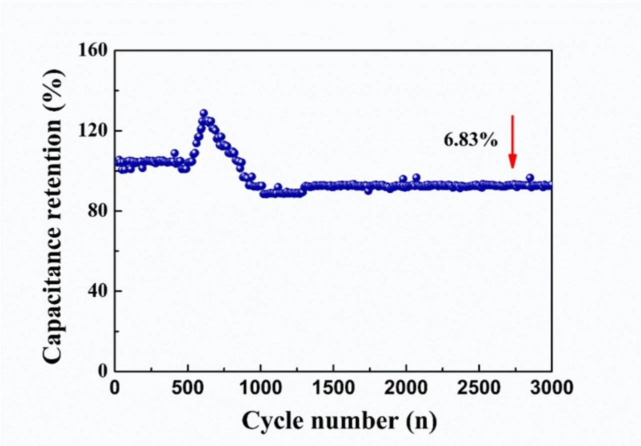

Fig. S4 The capacitance retention at a current density of 10 $A \cdot g^{-1}$ for 3000 cycles.

Table S1 Comparison of electrochemical performances of SCGN electrode in our work with previous.

Materials	Specific capacitance	capacitance retention (%)	Stability (%)	Current density	Refs.
Co_3O_4@MnO_2composites	671 F·g^{-1} at 1.0 A·g^{-1}	84% (1 to 10 A·g^{-1})	95.2% (2000 cycles)	1 A g^{-1}	1
Co_3O_4-CeO_2composites	1288.3F·g^{-1} at 2.5 A·g^{-1}	80.4% (1 to 6 A·g^{-1})	98% (6000 cycles)	4 A·g^{-1}	2
Co_3O_4/NF composites	2053.1 F·g^{-1} at 1.0 A·g^{-1}	54.2% (1 to 40·A g^{-1})	93.3% (10000 cycles)	2 A·g^{-1}	3
SmN-RGO composites	268 F·g^{-1} at 2.0 A·g^{-1}	----	99% (4000 cycles)	2 A·g^{-1}	4
$SmWO_4$ composites	326 F·g^{-1} at 2.0 mV·s^{-1}	47% (1 to 16 A·g^{-1})	89.9% (3000 cycles)	20A·g^{-1}	5
$SmCoO_3$/rGO composite	69.3 F·g^{-1} at 1.0 A g^{-1}	----	74.28% (15000 cycles)	1 A·g^{-1}	6
Sm_2O_3/Co_3O_4/rGO composites	3448 F·g^{-1} at 1 A·g^{-1}	44.8% (1 to 20 A·g^{-1})	93.2% (3000 cycles)	10 A·g^{-1}	This work

1. H. Che, Y. Lv, A. Liu, J. Mu, X. Zhang and Y. Bai, *Ceramics International*, 2017, **43**, 6054-6062.
2. C. Wei, K. Liu, J. Tao, X. Kang, H. Hou, C. Cheng and D. Zhang, *Chem Asian J*, 2018, **13**, 111-117.
3. G. Wei, L. Yan, H. Huang, F. Yan, X. Liang, S. Xu, Z. Lan, W. Zhou and J. Guo, *Applied Surface Science*, 2021, **538**, 14932.
4. A. S. Dezfuli, M. R. Ganjali and H. R. Naderi, *Applied Surface Science*, 2017, **402**, 245-253.
5. A. Sobhani-Nasab, H. Naderi, M. Rahimi-Nasrabadi and M. R. Ganjali, *Journal of Materials Science: Materials in Electronics*, 2017, **28**, 8588-8595.
6. M. Isacfranklin, R. Yuvakkumar, G. Ravi, S. I. Hong, D. Velauthapillai, M. Thambidurai, C. Dang, T. S. Algarni and A. M. Al-Mohaimeed, *Carbon*, 2021, **172**, 613-623.

References
